# Assessing the impact of harm reduction programs on law enforcement in Southeast Asia: a description of a regional research methodology

**DOI:** 10.1186/1477-7517-9-23

**Published:** 2012-07-09

**Authors:** Nick Thomson, Tim Moore, Nick Crofts

**Affiliations:** 1 Nossal Institute for Global Health, University of Melbourne, 4th Floor, 161 Barry St, Carlton, Victoria, 3010, Australia; 2 Johns Hopkins Bloomberg School of Public Health, Johns Hopkins University, Baltimore, USA; 3 Centre for Law Enforcement and Public Health, Melbourne, Australia

## Abstract

For over 15 years the Australian Agency for International Development (AusAID) has been a leading donor for harm reduction projects in Southeast Asia. The recent AusAID-supported harm reduction projects of greatest significance have included the Asia Regional HIV/AIDS Project (AHRP), from 2002 until 2007,1 and the HIV/AIDS Asia Regional Program (HAARP), from 2007 until 2015.2 Both projects included in their design specific strategies for engaging with law enforcement agencies at country level. The main focus of these strategies has been to develop law enforcement harm reduction policy and curriculum, and the design and implementation of specific harm reduction training for law enforcement officers.

In July 2008, the Australian Development Research Awards (ADRA) funded the Nossal Institute for Global Health at the University of Melbourne to establish a research project created to assess the influence of harm reduction programs on the policy and operational practices of law enforcement agencies in Southeast Asia, known as the LEHRN Project (Law Enforcement, Harm Reduction, Nossal Institute Project). The ADRA is a unique grant research mechanism that specifically funds development research to improve the understanding and informed decision making of the implementation of Australian aid effectiveness.

While the need to engage law enforcement when establishing harm reduction programs was well documented, little was known about the impact or influence of harm reduction programs on policy and practices of law enforcement agencies. The LEHRN Project provided the opportunity to assess the impact of harm reduction programs on law enforcement in Southeast Asia, with a focus on Vietnam, Cambodia and Lao PDR.

## Introduction

For over 15 years the Australian Agency for International Development (AusAID) has been a leading donor for harm reduction projects in Southeast Asia. The recent AusAID-supported harm reduction projects of greatest significance have included the Asia Regional HIV/AIDS Project (AHRP), from 2002 until 2007 [1], and the HIV/AIDS Asia Regional Program (HAARP), from 2007 until 2015 [2]. Both projects included in their design specific strategies for engaging with law enforcement agencies at country level. The main focus of these strategies has been to develop law enforcement harm reduction policy and curriculum, and the design and implementation of specific harm reduction training for law enforcement officers.

In July 2008, the Australian Development Research Awards (ADRA) funded the Nossal Institute for Global Health at the University of Melbourne to establish a research project created to assess the influence of harm reduction programs on the policy and operational practices of law enforcement agencies in Southeast Asia, known as the LEHRN Project (Law Enforcement, Harm Reduction, Nossal Institute Project). The ADRA is a unique grant research mechanism that specifically funds development research to improve the understanding and informed decision making of the implementation of Australian aid effectiveness.^a^

While the need to engage law enforcement when establishing harm reduction programs was well documented, little was known about the impact or influence of harm reduction programs on policy and practices of law enforcement agencies. The LEHRN Project provided the opportunity to assess the impact of harm reduction programs on law enforcement in Southeast Asia, with a focus on Vietnam, Cambodia and Lao PDR.

## Background and rationale

The HIV epidemic in Southeast Asia is largely a concentrated epidemic among key affected populations including people who inject drugs, men who have sex with men and sex workers [3]. In many countries across Asia the use of illicit drugs, sex between men and sex work are criminalised and this often brings people who engage in these activities into damaging interactions with law enforcement agencies. Harm reduction in the context of injecting drug use and HIV has been slowly evolving across countries in Southeast Asia but has not at this stage been able to reach coverage rates considered necessary to halt and reverse the spread of HIV. The inability to take harm reduction programs to scale is largely a result of being unable to resolve the tensions that exist between the policies and programs established to address HIV among people who inject drugs (PWID), on the one hand, and policies and approaches aiming to control or eradicate the availability and use of illicit drugs on the other. As a result of the latter, the law enforcement community often seeks to arrest, prosecute and detain PWID. Clearly, effective approaches to minimise HIV risk among and from PWID must occur within the context of supportive law enforcement policy and practice.

Law enforcement policy and practice is explicitly, if gradually, evolving towards greater endorsement of harm reduction approaches in Vietnam, Cambodia and Lao PDR. AusAID’s programs and complementary initiatives are occurring at a critical juncture to effect supportive and sustainable change to such policy and practice in the region, and thus allow and support the scaling up of efforts to prevent HIV among and from PWID.

The impacts – generally negative – of law enforcement policy and practice on HIV risk environments and behaviours and on harm reduction programs has been researched and documented, but little is known about how harm reduction programs impact law enforcement policy and practice in Asia. Are the programs and approaches (including advocacy) which are aimed at fostering an environment supportive of harm reduction for all people who used drugs, effective at influencing law enforcement policy and practice? The LEHRN Project was established to address this crucial knowledge gap by elucidating fundamental enablers and barriers to change within the culture of law enforcement agencies, and by key law enforcement actors, in various settings, from national to grass-roots level.

This paper describes the overall project methodology used to understand the impact of harm reduction programs on law enforcement policy and practice in Vietnam, Cambodia and Lao PDR.

## Research objectives

The LEHRN Project was aimed at addressing two main objectives:

1. To identify key drivers of the endorsement and incorporation of harm reduction principles in law enforcement policy and practice, in a variety of socio-political contexts, in three countries of Southeast Asia in order:

· To build a stronger evidence base to guide effective harm reduction policy and programs;

· To help create supportive environments for street-level harm reduction activities for women and men who inject drugs and;

· To provide practical, field-derived and tested recommendations for AusAID and other agencies implementing harm reduction programs in Southeast Asia in regard to establishing positive collaboration with law enforcement.

2 To strengthen the regional capacity of relevant researchers and institutes in Asia, and of existing agencies, forums and networks, to understand and work more effectively with law enforcement in the context of HIV prevention and harm reduction programs working with PWID.

In working towards the study objectives, the project employed three distinct phases implemented over a 4-year time period. Phase one included a conceptual study design phase, the building of the study infrastructure and conducting the background contextual analysis. Phase two involved the collection of primary data and an initial country level analysis. The third phase involved a more in-depth comparative analysis of the results from each of the three countries, the write up of papers and the dissemination of the research findings. The following section describes these phases and comments on the limitations and challenges that resulted and adjustments that were made in order to adapt to the different country contexts.

## Phase one: study design, infrastructure building and background contextual analysis

The research design used qualitative methods to map the associations between various harm reduction approaches and changes in formal/informal law enforcement policy and practice. The study was designed with the expectation that at the local level the researchers would form working relationships with law enforcement actors and use semi-structured interviews and observational techniques to understand the law enforcement cultures, practices and influences. It was expected that detailed examples of how law enforcement policy and practices were influenced by harm reduction programs and policies could be identified. It was further expected that through analysis of interviews, observations and case studies, pathways and determinants of change in policy and practice of law enforcement could be produced to identify patterns of association.

The design was underpinned by a conceptual framework that recognised: (1) there are often contradictions between policy and practice and each may influence the other; (2) the pace of policy change is often uneven; (3) formal policy development often occurs subsequent to ‘on the ground’ experience, and responds to pragmatic imperatives; and (4) different levels of government (e.g., national, district) can influence policy at other levels. Given the vastly different contexts of drug use, policing and harm reduction programs in each of the selected countries, the study design was not expected to be identical in all countries. Using qualitative approaches offered advantages not only for flexible exploration of local dynamics, but allowed for adaptation to local conditions.

### Selection of field sites

The three countries were selected to provide a diversity of settings in relation to drugs, HIV and policy development that would enable comparisons to be drawn. Vietnam was directly involved in AusAID’s ARHP, and all three subsequently, during the course of the LEHRN Project, in HAARP, thus conferring potential for rapid uptake of research findings. The countries were further selected as LEHRN Project team members from the Nossal Institute had several affiliates stationed in Southeast Asia with multi-country experience in working in HIV projects and facilitating research in partnership with national institutions.

At the time of the study design, the following country specific details were considered relevant: Vietnam had a high prevalence of both illicit drugs and of HIV among PWID a long exposure to harm reduction approaches; a nationally endorsed harm reduction policy, had implemented programs to varying degrees of scale and had been a recipient of AusAID harm reduction programs since 2002. Cambodia had a relatively high HIV prevalence among the general population that has fallen; was in the midst of an early and rapid rise in injecting drug use and of HIV among injecting drug users; and a good basic policy that was early in development. The Lao PDR was considered a low prevalence country for both injecting drug use and of HIV; was very early in the policy development cycle of harm reduction; and was a recipient of development assistance for harm reduction programs post-opium eradication.

### Research infrastructure and partnerships

During this phase several site visits were made by the Principal Investigator and project staff from the Nossal Institute to strengthen relationships with partner institutes in the three countries to develop and ratify Letters of Understanding governing the research and capacity-building relationships and communication strategies. The Nossal Institute sought Country Research Partners with strengths in social and public health research and potential to contribute to national policy development. The selected partner for Vietnam was the Institute for Social Development Studies (ISDS), an institute with sound experience in research, training and advocacy on gender and sexuality, health policy development, HIV and social development. The ISDS had recently undertaken research titled *Understanding subcultures of drug users for HIV interventions*. For Cambodia, the Nossal Institute established collaboration with the National Institute of Public Health (NIPH), a semi-autonomous institution located within the Ministry of Health (MOH). The NIPH is considered influential in determining health policies and direction through evidence-based research provided to Ministry of Health. In Laos PDR, the Nossal Institute identified the University of Health Sciences as the preferred partner for its good track record in social research and potential to influence national drugs and HIV policy.

The Principal Investigator and key team members from the Nossal Institute brought to the LEHRN Project intimate understanding of issues around injecting drug use and HIV responses in Southeast Asia. This was complemented by the activation of a reference group, of particular importance in Phase one, comprising noted academics from the University of Melbourne, Macquarie University and Johns Hopkins/Chiang Mai Universities. The reference group members’ expertise included research methodology, gender, policy development, drug and law enforcement culture, and anthropology, public health and harm reduction. A further and crucial addition to the core project team was a postgraduate Research Fellow, who was also an active constable of the Victoria Police (Australia), and who undertook an advanced policy design course once engaged.

### Regional workshops, trainings and research team communication

After partner selection, a regional workshop in Hanoi brought together key primary team members, including Country Research Partners, as well as regional collaborators including AusAID staff. The workshop helped to cement the wider LEHRN Project team and provided an opportunity for further discussions on the research design, the training requirements and the ethic approval processes. This was the first of five project regional workshops facilitated during the life of the project.

### Background situational analysis

Phase one involved a background situational analysis upon which to contextualise the research in each country and create a baseline from which the influences over time of harm reduction programs on police policy and practice could be assessed. The background situational analysis was underpinned by a comprehensive desk review. In conjunction with the Country Research Partners the project team collected relevant country level policy documents and literature, reviews of existing studies and donor project evaluations that had generated data or recommendations on working with police and regional/country-level harm reduction programs to understand any relevant initiatives that were currently operating. The LEHRN team attempted to develop an understanding of the mechanisms of policy development and policy review across the law enforcement and health sectors and how this applied to the intersection of police and public health policies and practice in relation to harm reduction, HIV and injecting drug use in each of the three countries. By examining the theoretical determinants of positive change to policy and practice within given law enforcement cultures and settings, the policy review process was originally designed to generated hypotheses of the impacts of harm reduction strategies and advocacy on the harm reduction and law enforcement nexus that could be tested through primary data collection phase.

## Phase two: stakeholder mapping, primary qualitative data collection and preliminary analysis

Phase two of the project comprised stakeholder mappings, finalisation of qualitative data collection tools, primary data gathering and preliminary analysis. After analysis of the background context of each country, project team members from the Nossal Institute travelled to each country in order to finalise the qualitative data collection tools and decide upon a sampling framework for recruitment of participants to conduct interviews. In order to finalise the qualitative interview guides, the research teams undertook a stakeholder mapping exercise to better understand how each country’s law enforcement sector was structured.

### Stakeholder mapping

Detailed national-level mappings of protagonists within law enforcement agencies, and actors positioned sufficiently closely to them to be of influence or make astute comment thereof, constituted a major component. In addition, the research teams gave thought to where harm reduction programs would intersect with police and where decisions around police responses to harm reduction would be made. This mapping was a critical step to identify chief interviewees and focal points for this phase of the project.

### Qualitative data tool development

The original conceptual study design was based upon interviewing key informants to provide the basis for an inductive approach to identification and description of policy and decision-making networks and influential processes. Primary data was to not only be collected using semi-structured interview guides with key informants, but also through semi-structured observation of the relationship between relevant sectors and consistency between explicit policy and grass-roots practice. These approaches were designed to enable the mapping of key nodes in policy development and would potentially result in the ability of the research to map networks of influence, both formal and informal, of government as well as non-government and private sectors.

The original research design proposed to ask law enforcement officials: “Who is important to you, within your professional network, regarding policy and practice?” It was further proposed that key informant interviews with regional and in-country stakeholders would explore the links between harm reduction programs and law enforcement policy and practice. Stakeholders that the original research design proposed to interview included law enforcement representatives, MOH/MOPH, AusAID HIV project offices, UN agencies, people who used drugs, relevant NGOs/CBOs.

During the background analysis and stakeholder mapping exercise, it became clear each country was at a very different stage of the development of harm reduction policies and programs. In addition, Country Research Teams had very different perceptions about their abilities to interview the various levels of police and other law enforcement protagonists identified by the stakeholder mapping. The resulting primary data collection tools were a reflection of both where the country was in its response to drug use and HIV, and the various power dynamics between researchers and law enforcement officials. The resulting set of semi-structured interview guides for both key informants and participants are described more completely in the country specific papers.

### Regional workshops, trainings and research team communication

The second regional workshop was held in Bangkok alongside the ‘Harm Reduction 2009: Harm Reduction International’s 20th International Conference’, at which LEHRN Project team members also presented.

Early in Phase two it became apparent, in order to consolidate each of the country level background analyses and stakeholder mappings, initiate the development of papers for publication, to bolster confidence and approaches to undertaking research in the politically charged environment of law enforcement and HIV, clarify issues of project administration and forward planning, and generally instil a spirit of collegiality among the project staff especially the Country Research Partners, extended time together was necessary. As such, all partners were brought together for three weeks in Melbourne with the Nossal Institute, toward the end of 2009. A seminar on “Law Enforcement and Harm Reduction in Southeast Asia” was held during that time to coincide with the partners’ visit, for which LEHRN Project representatives from each of the three nations gave presentations to a diverse audience.

In February 2010, the LEHRN Project hosted a fourth workshop, this time in Phnom Penh, which included training in NVivo software (see below). This was one of three back-to-back events. The second was the seminar on “Law Enforcement and Harm Reduction: Effective Partnerships” for which, once again, the LEHRN Project delivered several presentations to a local and regional audience including Cambodian police representatives. The closing event was the “Roundtable Discussion on Law Enforcement and Harm Reduction in Cambodia”. The outputs of this event included a first set of recommendations about engaging law enforcement in the harm reduction enterprise and are outlined below [4].

### Policing and harm reduction – principles of collaboration

Major points highlighted by the LEHRN Seminar, Phnom Penh, Feb 2010:

1. The involvement of law enforcement is critical to the success of harm reduction programs at all levels – regional, national and local.

2. There is a pressing need for law enforcement agencies and authorities to share ownership of harm reduction.

3. Police must be engaged early by harm reduction programs; not as a subsidiary but as a core partner.

4. There is a need to document the experiences of law enforcement and harm reduction working together in the region, at all levels, both positive and negative.

5. Involvement of law enforcement at local level must be through effective community partnerships based on mutual understanding and respect, and should include local communities, local police authorities and other relevant partners.

6. There need to be multi-sectoral structures among all key agencies involved at all levels, so that working relationships can be established and maintained.

7. To ensure police and others in the law enforcement sector are enabled to fulfil a harm reduction mission and have the capacity to be effective partners, they need adequate resourcing.

8. Harm reduction activities must be integrated into police planning, and show congruency with other government department plans.

9. Political awareness and support are fundamental to the success of law enforcement and harm reduction partnerships and programs, and must be matched by government leadership and investment in harm reduction.

10. Solutions must be practical and be seen to be of worth by police – police responses and responsibilities in the partnership must be operationalised.

Later that year, the LEHRN Project gave presentations at the “Health and Human Rights Conference” in Hanoi.

### Preliminary analysis

As described, the research teams in each country were introduced to the use of the NVivo software to analyse qualitative data in a training led by ISDS staff members expert in using this software package. The training provided opportunity to share and reflect on the preliminary data. In particular, the LEHRN Project team discussed and systematically collated the themes that were emerging. Thus a comprehensive “node tree” or well-populated thematic clusters were established to enable Country Research Teams to code and analyse the qualitative data being collected. These clusters fell within several broad categories: “harm reduction – evidence”; “harm reduction – programs and services”; “means of influence”; “law enforcement/police – policy”; law enforcement/police – practice”; and “other factors”.

### Case studies

The original study design had planned on tracking national case studies following the documented development of and changes in law enforcement policy and practice related to illicit drugs and harm reduction. The case studies were to be supported by the conducting of supplementary key informant interviews and participant observation of police practice and attitudes. Given the often closed and opaque cultures of law enforcement in the selected research countries, it became clear that our research teams would be unlikely to be able to conduct this level of observation. The research team instead decided to support the key informant and participant interviews with follow up case studies on country specific topics of interest. As such, relevant and complementary studies emerged for Vietnam, Lao PDR and Cambodia that highlight unique insights into particular intersections of law enforcement and harm reduction programs or practices and provide contextual examples which reinforce the findings reported in the country papers.

## Phase three: comparative analysis, country research papers and a dissemination strategy

Upon completing the interviews and conducting a preliminary analysis, each country team drafted a research report and paper. These papers were also worked on by project staff from the Nossal Institute and sent for peer review. As the sampling framework of key informant interviews was different in each country, it became clear that a comparative analysis using NVivo software would likely prove difficult. The research team instead opted for a descriptive comparative analysis. Regional and country-level forums were held to disseminate findings to key stakeholders. The dissemination workshops in each country followed a regional launch of the monograph in Bangkok in the first week of July 2012. The regional launch and country level disseminations were conducted with collaborative inputs and support from the AusAID Regional Office in Bangkok and were designed as advocacy opportunities to highlight vital strategies and important considerations for harm reduction program design and implementation in the context of the need for partnership development with law enforcement agencies working at both the political and program level in Vietnam, Cambodia and Laos PDR.

## Discussion of methodology

This multi-country research project was the first of its kind to examine the role and influence of harm reduction programs on law enforcement policy and practice with regards issues of illicit drug use and HIV. It was hoped that we would be able to produce models of ‘good’ harm reduction and law enforcement outcomes and to a certain degree the series of papers and its concluding and comparative analysis has been able to highlight set principles and strategies by which harm reduction programs can work more effectively with law enforcement. As an exercise in conceptual research design which leads to on-the-ground implementation, it has been also been a very informative exercise that warrants further reflection.

The LEHRN team set out to document real-time changes in law enforcement policy and practice over a two-year data collection period. It was intended to get serial interviews to ascertain how key informants and agencies responded to expansions in harm reduction programs. Although this happened to some extent, unearthing real-time changes proved somewhat difficult in Cambodia and Lao PDR, particularly as the evolution of harm reduction in these contexts is complex and often opaque. Furthermore the complexity of the nexus of harm reduction and law enforcement in each country impacted the capacity of research teams to conduct this type of research, in an operational sense, in what is a very narrow political space. This became relevant in the analysis and understanding of the relationship, and therefore potential influence, prominent members of the health and academic sectors in each of the three countries could have with and on law enforcement.

Our original conceptual research was designed before the project had fully engaged at country level with our Country Research Partners. The design was complex and aspirational and had the team tracking shifts in regional and country level harm reduction initiatives, and national law enforcement policy and practices, measured against set baseline indicators and assessed using theories of law enforcement and health sector policy development across each country. The aim was to conduct comparative case studies grounded in primary data that could be compared by identifying natural experiments demonstrating before and after change, differences between comparable settings, and so forth.

This work would have involved making the models specific to the context, with identification of particular drivers and modifiers of policy and practice from the data.

In reality, we were able to implement the research design much as originally planned, but not to the level of sophistication envisaged at the outset. In order to accomplish research objectives, the team needed to be sufficiently flexible and adaptable, moving within the design framework and maintain a realistic outlook regards research outputs. Viewed from that perspective, research efforts have been successful and the project has indeed been able to show the two-way relationship between policy and practice in relation to law enforcement and harm reduction programs, and the need for meaningful relationships between law enforcement and harm reduction programs to produce better outcomes for people who use drugs and their communities in terms of HIV.

Moreover, the ADRA grant mechanism is not only aimed at answering research questions: equally it is about capacity development. That is, the ADRA model is not purely about research and it is not just a project. Unequivocally, the LEHRN Project built a close network of country teams in Australasia, confident and capable of continuing to work on cutting-edge research at the intersection of complex issues around law enforcement and public health.

## Endnotes

^a^ More information on the ADRA mechanism is available at http://www.ausaid.gov.au/research/default.cfm. Accessed on 17 January 2012.

## Competing interests

The authors declare that they have no competing interests.

## Authors’ contribution

All authors were involved in the writing of this manuscript. TM and NC were responsible for the original study design. TM and NC were also primarily responsible for the training workshops described in this manuscript. NT and TM were responsible for drafting the original version of this manuscript. TM and NC were responsible for revising the manuscript into its current form. All authors read and approved the final manuscript.
